# Effects of rainfall, temperature and topography on malaria incidence in elimination targeted district of Ethiopia

**DOI:** 10.1186/s12936-021-03641-1

**Published:** 2021-02-19

**Authors:** Desalegn Dabaro, Zewdie Birhanu, Abiyot Negash, Dawit Hawaria, Delenasaw Yewhalaw

**Affiliations:** 1 Yirgalem Hospital Medical College, Yirgalem, Ethiopia; 2 Department of Medical Laboratory Sciences and Pathology, College of Health Sciences, Jimma University, Jimma, Ethiopia; 3 Department of Health, Behaviour and Society, Faculty of Public Health, Jimma University, Jimma, Ethiopia; 4 Tropical and Infectious Diseases Research Center, Jimma University, Jimma, Ethiopia; 5 Department of Statistics, College of Natural Science, Jimma University, Jimma, Ethiopia

**Keywords:** Malaria, Temperature, Rainfall, Altitude, Boricha, Ethiopia

## Abstract

**Background:**

Climate and environmental factors could be one of the primary factors that drive malaria transmission and it remains to challenge the malaria elimination efforts. Hence, this study was aimed to evaluate the effects of meteorological factors and topography on the incidence of malaria in the Boricha district in Sidama regional state of Ethiopia.

**Methods:**

Malaria morbidity data recorded from 2010 to 2017 were obtained from all public health facilities of Boricha District in the Sidama regional state of Ethiopia. The monthly malaria cases, rainfall, and temperature (minimum, maximum, and average) were used to fit the ARIMA model to compute the malaria transmission dynamics and also to forecast future incidence. The effects of the meteorological variables and altitude were assessed with a negative binomial regression model using R version 4.0.0. Cross-correlation analysis was employed to compute the delayed effects of meteorological variables on malaria incidence.

**Results:**

Temperature, rainfall, and elevation were the major determinants of malaria incidence in the study area. A regression model of previous monthly rainfall at lag 0 and Lag 2, monthly mean maximum temperature at lag 2 and Lag 3, and monthly mean minimum temperature at lag 3 were found as the best prediction model for monthly malaria incidence. Malaria cases at 1801–1900 m above sea level were 1.48 times more likely to occur than elevation ≥ 2000 m.

**Conclusions:**

Meteorological factors and altitude were the major drivers of malaria incidence in the study area. Thus, evidence-based interventions tailored to each determinant are required to achieve the malaria elimination target of the country.

## Background

The burden of malaria has been reduced globally due to the wide-range implementation of multiple interventions. The number of malaria cases and deaths had declined by 18% and 48% between 2000 and 2015. Similarly, the incidence and the mortality rate, both used the population growth into consideration, had declined by 37% and 60% in the same period. From 2000 to 2017 alone, 20 countries have eliminated malaria transmission and reported zero case for at least one year. These successful signs of progress of malaria control have prompted the worldwide possibility of malaria elimination. As a result, the World Health Organization (WHO) has developed a global malaria elimination programme in 2015 intending to make a malaria-free world by 2030. Currently, the elimination programme is under implementation in many countries including Ethiopia [1–4].

Notable progress has been made since the elimination programme was launched worldwide. Globally, the number of countries reporting below 10,000 cases and below 100 indigenous malaria cases increased from 40 to 49 and 17 to 27, respectively, from 2010 to 2018. From 2017 to 2018 alone, the number of countries reporting below 10 indigenous cases increased from 19 to 24. As a result, 19 countries had zero indigenous cases for 3 and above successive years from 2000 to 2018, while 4 countries, which were malaria endemic in 2015, attained malaria elimination. The burden of the disease has substantially declined in Africa, including Ethiopia, the continent most affected [1–3, 5, 6].

Climate change and geographical elevations are among the major factors affecting transmission and the geographical distribution of malaria. Meteorological variables, such as rainfall, temperature and humidity could impact the bionomics of malaria vectors, which could eventually determine malaria transmission intensity. Several studies reported the controversial association between climate change and malaria transmission [7–9].

Moreover, a higher elevation is another determinant of malaria incidence and transmission. As the altitude increases the temperature decreases and vice versa that in turn influences the transmission dynamics of the infection. Besides, the geographical shift of the *Anopheles* mosquito due to climate change facilitates malaria transmission in previously non-malaria areas, which results in an uneven distribution of the disease [10–16]. The effect of climate varies with different agro-ecological areas. Therefore, malaria prediction models using meteorological data vary from place to place thus only a single model could not fit for all geographical locations. Such inconsistencies of predictions are due to variations of risk factors of malaria incidence [17–19].

Ethiopia has unstable and highly seasonal malaria incidence with varying intensity of transmission depending on the climatic variations and geographical settings of the country [20]. Thus, the current study was aimed to investigate the impact of climate and elevations on malaria transmission in Boricha district, one of the malaria-endemic and elimination targeted districts in the country.

## Methods

### Study area and population

The study was conducted in Boricha district, in the Sidama regional state of Ethiopia; located at 304 km from the capital city of Ethiopia, Addis Ababa. It covers a total area of 588.1 square kilometres. The altitude of the district ranges from 1001 to 2076 metres above sea level. The mean annual rainfall of the district ranges from 801 to 1000 mm, and the mean annual temperature ranges from 17.6 to 22.5 °C [21]. The district has 42 *kebeles* (the lowest administrative unit), 39 rural and 3 urban, and has a total populations of 325,161. Most of the population live in rural areas where agriculture is the mainstay of the community [22, 23].

The district is one of the malaria-endemic districts in the region. Therefore, several interventions have been implemented to prevent and control the disease in addition to prompt diagnosis and treatment. The diagnosis and treatment services have been given in both public and private health facilities. The public health facilities include one primary hospital, ten health centres, and thirty-nine health posts, and the private clinics including one non-governmental clinic, one medium clinic, five primary level clinics, three drug stores, and eleven drug vendors [23].

### Study design

Retrospective malaria morbidity data were collected from health facilities to determine the association between meteorological variables and topography with malaria incidence over the last eight years (2010–2017) in Boricha district.

### Source of information

The source of information was the malaria laboratory register logbooks at malaria diagnosis and treatment services providing health facilities in the district. The meteorological data of the district were obtained from the National Meteorological Agency (NMA) of the country [24].

### Malaria diagnosis

In the study area, diagnosis of malaria was performed based on the national malaria guideline, which recommends both clinical and parasitological (laboratory) diagnosis of the disease in health facilities. A clinical diagnosis was performed based on the patient’s history. However, a clinical diagnosis alone was not recommended except when there was no Rapid Diagnostic Test (RDT) or light microscopy in the health facilities. Parasitological (laboratory) diagnosis was performed using RDTs or light microscopy to confirm malaria parasites. Both thick and thin blood films were prepared to detect the malaria parasites. similarly, RDT was used in health posts to enhance malaria diagnosis at the periphery level [25].

### Data collection techniques

In the current study, only laboratory-confirmed malaria cases were used. As a result, the laboratory registers of malaria morbidity were collected from all health facilities, which provide malaria diagnosis and treatment services. From which, laboratory-confirmed malaria cases were obtained from one primary hospital, nine health centres, and thirty-seven health posts. The rest facilities were newly established and not fully equipped. As a result, it had been providing only the clinical diagnosis of malaria due to the absence of a light microscope and RDTs.

Meteorological data of the district, such as the minimum temperature, maximum temperature, average temperature, monthly rainfall were obtained from the NMA of Ethiopia [24]. The geographical coordinates of each kebele were recorded by geographical positioning system using Garmin eTrex 10 device. The data of kebele coordinates were collected by one trained data collector, where the location of the health facilities, mainly health post in each kebele was taken as a reference point of measurement.

The records of laboratory registers were collected by trained laboratory technicians using a standard format adapted from the national laboratory malaria register (logbook). Only the complete records, including the date (year and month) of diagnosis, address of the patients, age, sex, and the results of the diagnosis were included in the study. Incomplete records were excluded because each variable was required for analysis. Malaria reported from the study district only were used in the study, and the cases reported from out of the study area were also excluded. The year and month of the diagnosis were required to compute the trends and seasonality of malaria incidence. The address of the patients was required to link the cases with their specific residence, which was useful to understand the geographical variations of malaria distribution at several elevation levels. Age and sex were also analysed to understand the segments of the population more affected. The whole process, data retrieving and data entry in computer software were highly supervised to ensure completeness and consistency.

### Data analysis

Data were entered using EpiData 3.1 and analysed using R version 4.0.0. Different statistical models were developed and used to predict the incidence and transmission patterns. Auto-regressive integrated moving average (ARIMA) model was used on monthly time series of malaria morbidity. The general linear model (GLM), negative binomial and Poisson regression, was used to compute the pattern of malaria distribution in the district. Auto-correlation Function (ACF) and partial autocorrelation function (PACF) of the residuals and the Ljung-Box Q test were used for the model adequacy checking. The upward trends of malaria transmission in the district, which could highly explained by the environmental factors, were determined using the ARIMA model.

### Data quality control measures

Data collectors, supervisor and data entry clerks were trained on the objective and significance of the study. Also, data collectors and supervisor were trained about the data collection tools, and data entry clerks were trained about the data entry software (EpiData 3.1). In general, training was given for two days, one day for data collectors and supervisor, and one day for data entry clerks. The completeness and consistency of the data were strictly followed by the supervisor of the data collection. Also, the principal investigator has been following the whole process, such as completeness and consistency of collected data and entry software as well to enhance the quality of data.

## Results

### Descriptive statistics of malaria

A total of 28,413 confirmed malaria cases were recorded over eight years, from 2010 to 2017 in the study area. From which, adults 15 years and above age category were 14,722 (51.8%) while children 5–14 years and < 5 years age groups were accounted for 6944 (24.4%) and 6,747 (23.8%), respectively. A relatively high proportion, 14,575 (51.3%) of cases were males. In general, malaria occurred in all months of the year throughout the study period where a minimum and maximum monthly case number was 3 and 859, respectively where the mean monthly case number was 296 (66.77%).

### Time series plot of malaria

**Fig. 1:**
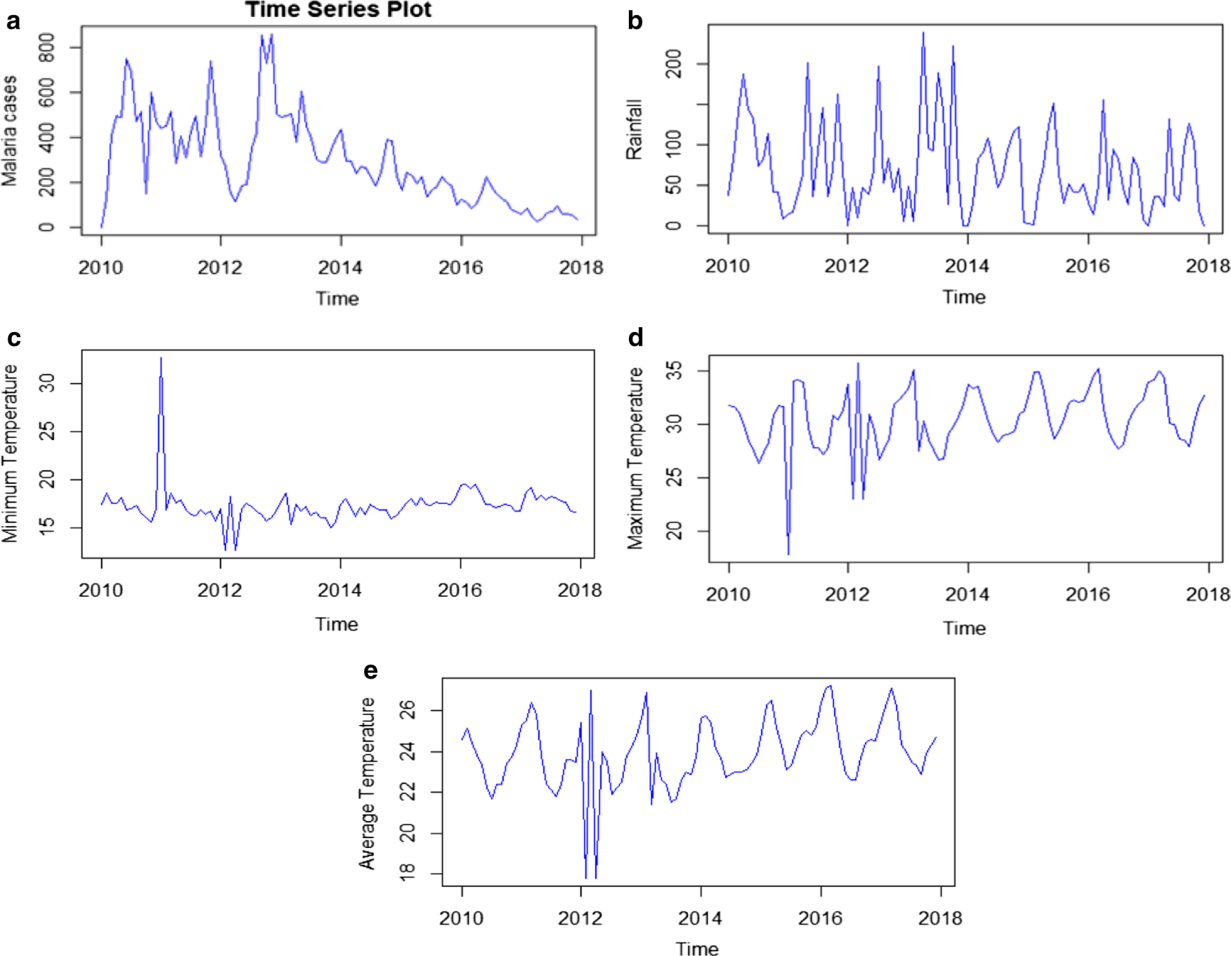
Time series plots of monthly malaria cases and meteorological variables in Boricha district in Sidama regional state, Ethiopia; **a** (Malaria cases), **b** (Rainfall), **c** (Minimum temperature), **d** (Maximum temperature) and **e** (Average temperature)

The time series plot of malaria cases shows a clear downward trend of malaria in eight consecutive years from 2010 to 2017. Here, successive observations (months) revealed a steady reduction of malaria incidence over the eight years. Besides, there was an identical pattern of trends of monthly malaria occurrence in each year indicating that the relative amplitude of seasonal changes was constant over time (Fig. 1a).

### Descriptive statistics of meteorological variables

Table 1 shows descriptive statistics of monthly recorded rainfall and temperature during the study period where a minimum and maximum amount of mean monthly rainfall was 0 and 238 mm, respectively. Similarly, the mean minimum and maximum values of monthly average temperature were 17.8 and 27.2 °C, respectively.

**Table 1:** Mean monthly rainfall and temperature in Boricha district in Sidama regional state, Ethiopia

Variable	Minimum	Maximum	Mean	CV (%)
Rainfall	0	238	70	78.53
Min.Temperature	12.70	32.70	17.32	11.25
Max.Temperature	17.8	35.7	30.46	9.65
Average Temperature	17.8	27.2	23.92	7.06

### Time series plots of meteorological variables

The series plot of rainfall revealed that the amount of rain was somehow constant overtime with some heavy downpours observed in 2013. The amount of high rainfall in a given period did not cause an immediate increment of malaria incidence. In general, several peaks and fluctuations in the series plot indicate the seasonal dependency of rainfall in the study area. Similar to rainfall, temperature exhibits cyclic patterns of occurrence indicating a seasonal dependency (Fig. 1b, e).

### Model comparison for malaria trend analysis

Four models were compared to analyse the nature of the trends describing malaria incidence. Accordingly, the Log-quadratic model was the best fit model based on the selection criteria due to the lowest Akaike’s Information Criteria (AIC) and Bayesian Information Criteria (BIC) (Table 2).

**Table 2:** Model comparison for the trend analysis of monthly malaria incidence in Boricha district in Sidama regional state, Ethiopia

Model	AIC	BIC
Linear	929.69	936.57
Quadratic	926.31	935.47
Log-linear	81.05	87.92
Log-quadratic^a^	68.28	77.44

^a^Means the best model based on selection criteria

To investigate the effect of seasonality, the logarithmic transformed malaria incidence data were regressed on the quadratic trend and seasonal dummies. The results indicate that the model was significant at p < 0.05 level (*F*-statistic = 548.7, p < 0.0001, R-square = 98.94%; and adjusted R-square = 98.76%) (Table 3).

**Table 3:** Estimates of the log-quadratic model with seasonal effects on malaria incidence in Boricha district in Sidama regional state, Ethiopia

Variable	Coefficient	Standard error	p-value
Trend	0.03638	9.058e–03	0.000131
Trend square	− 0.0005767	9.043e–05	1.01e–08
January	4.845	2.752e–01	< 2e–16
February	5.346	2.765e–01	< 2e–16
March	5.333	2.777e–01	< 2e–16
April	5.147	0.2788	< 2e–16
May	5.448	2.799e–01	< 2e–16
June	5.493	2.808e–01	< 2e–16
July	5.596	2.817e–01	< 2e–16
August	5.579	2.825e–01	< 2e–16
September	5.623	2.832e–01	< 2e–16
October	5.544	2.839e–01	< 2e–16
November	5.774	2.845e–01	< 2e–16
December	5.468	2.850e–01	< 2e–16

To measure the monthly changes, the data of malaria cases were first transformed and then regressed on the square trend and monthly dummy variables. The month of November has approximately 5.774 more malaria cases than other months suggesting that it is the month in which the highest number of malaria cases occurred. The least number of malaria cases occurred in April and January (Table 3).

The data were stationary (Augmented Dickey-Fuller Test, p = 0.01) time series. The results showed that the model that best fits the original data were ARIMA (2,1,2) (Table 4).

**Table 4:** Parameter estimates of the ARIMA (2,1,2) Model

Parameter	Estimate	Std. error	*Z*-statistic	Confidence interval	
ar1 (*ϕ*)	− 0.29	0.11	− 2.51	− 0.51	− 0.06
ar2 (*ϕ*)	0.68	0.10	6.25	0.47	0.90
ma1 (θ)	0.02	0.08	0.24	− 0.13	0.17
ma2 (θ)	0.87	0.07	− 12.49	− 1.00	− 0.73

Hence, the model for predicting future malaria case was ${Z}_{t}={X}_{t}-{X}_{t-1}$ and the model obtained was; ARIMA (2,1,2): ${X}_{t}={\alpha }_{1}{X}_{t-1}+{\alpha }_{2}{X}_{t-2}+{\beta }_{1}{Z}_{t-1}+{\beta }_{2}{Z}_{t-2}+{\epsilon}_{t}$; where, $\alpha ,\text{a}\text{n}\text{d} \, \beta$ are parameters and ${\epsilon}_{t}$ is the residual term. The substituted estimates of the parameters obtained were; ARIMA (2,1,2):

$${X}_{t}=-0.2884{X}_{t-1}+0.683845{X}_{t-2}+0.018611{Z}_{t-1}+0.867888{Z}_{t-2}+{\epsilon}_{t}$$

The final estimate of parameters for the model showed that the AR (1), AR (2), and MA (2) parameters had a *p*-value below 0.05, indicating a significant model parameter (Table 4).

### Cross‐correlation analysis of meteorological variables and malaria incidence

**Fig. 2:**
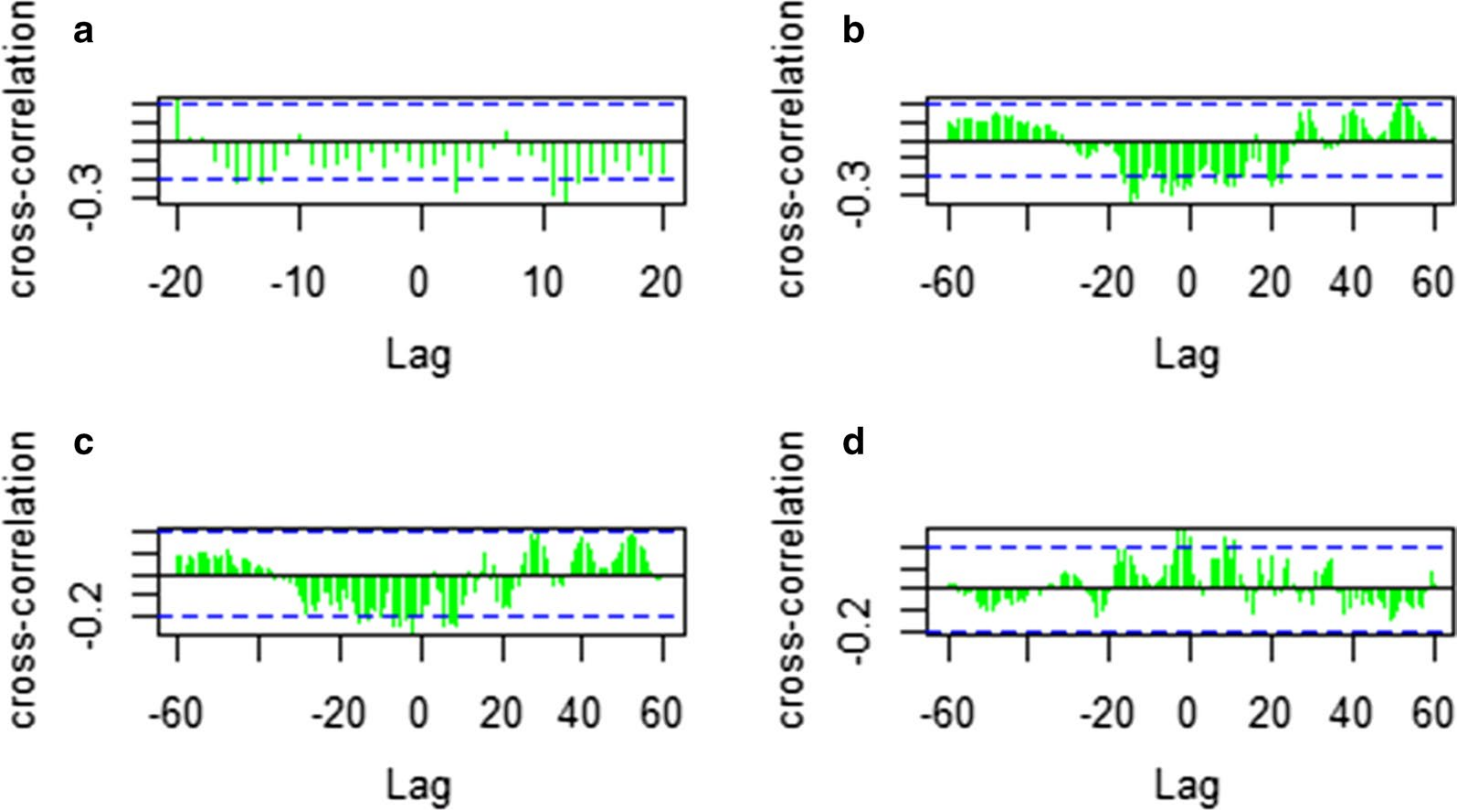
Cross-correlation functions of mean minimum temperature (**a**), Average Temperature (**b**), mean Maximum Temperature (**c**), and Rainfall (**d**)

Cross-correlation analysis showed that minimum temperature, maximum temperature, average temperature and rainfall have significantly lagged correlations with malaria case occurrence (Fig. 2). Cross-correlation analysis of rainfall shows a positive correlation with malaria cases (Fig. 2d). A regression model on lag 6, 7, 12, 13, and 15 of previous monthly average temperature, lag 3 and 11 of previous monthly mean minimum temperature, lag 2 and 3 of previous monthly mean maximum temperature and lag 0, 2 and 8 of previous mean monthly rainfall were found as the best prediction model for monthly malaria incidence. The monthly mean minimum, mean maximum and average temperatures were the leading and monthly mean rainfall was the lagging variable of the monthly malaria incidence.

**Fig. 3:**
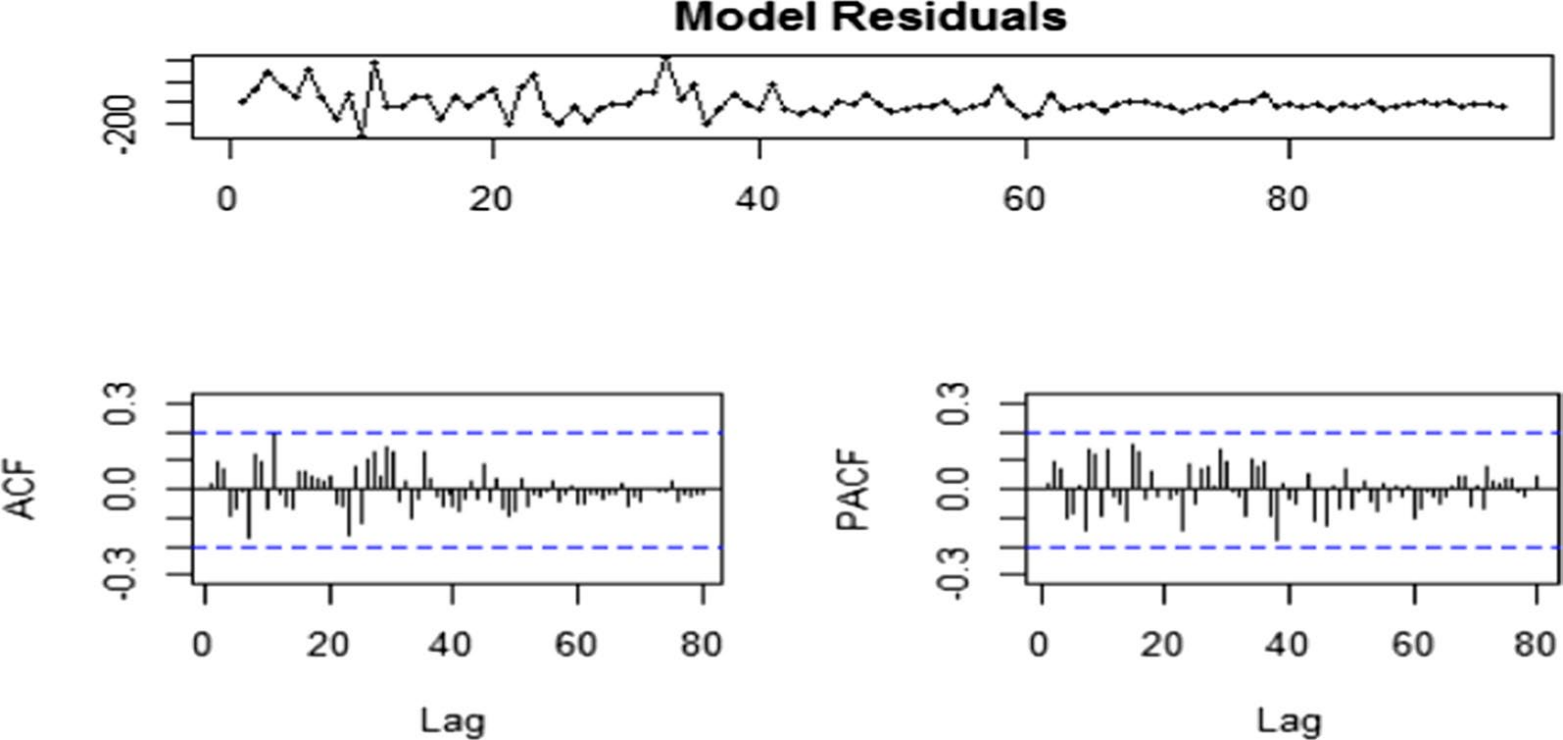
Diagnostic checks of ARIMA (2,1,2) residuals. Time series of residuals, the Autocorrelation function of residuals, Partial autocorrelation function of residual

The auto-correlations and PACF do not exceed the significant bounds, which was good. Using the Ljung-Box test there was no significant (p = 0.9927) auto-correlations between successive forecasting errors. The Ljung-Box test yielded a p-value of more than 0.05 indicating that the model was free from serial correlation. The values were normal as they rest on a line and were not all over the place. All the graphs are in support of the assumption that there was no pattern in the residuals and hence calculated the forecast while the model residuals were homoscedastic (Fig. 3).

**Fig. 4:**
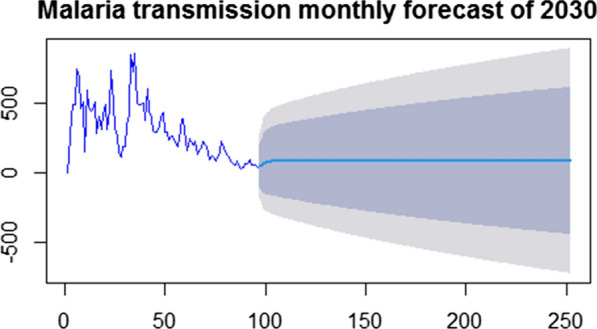
Malaria incidence monthly forecast for 2030

Forecasts (Fig. 4) based on an ARIMA (2,1,2) model in 2030 in November and December months, the malaria incidence were expected to be 88.367 and 88.281, respectively, which was approximately constant over all the year. The mean and variance for the malaria incidence were 1.17 and 5.17, respectively, with the ratio of variance to mean 4.40. The Poisson model is often criticized for its restrictive property that the conditional variance equals the conditional mean. Real-life data are often characterized by over dispersion that is the variance exceeds the mean. The negative binomial regression model is a generalization of the Poisson regression model that allows for over dispersion by introducing an unobserved heterogeneity term for observation. For this, the negative binomial model is recommended to model the data. Thus, Table 5 proved this since AIC and BIC for the Negative Binomial model are less than the Poisson regression model, which implies Negative Binomial is best to account for the dispersion.

**Table 5:** Poisson and negative binomial regression analysis and model comparison

Parameter	Poisson regression		Negative Binomial	
	Estimate (std. error)	P-value	Estimate (Std. Error)	P-value
Intercept	2.0412050 (0.1071191)	< 2e–16	2.5712491 (0.2180926)	< 2e–16
Month August	0.1997012 (0.0321717)	5.39e–10	0.1489509 (0.0596761)	0.01256
Month December	0.5959069 (0.0338554)	< 2e–16	0.6129467 (0.0621383)	< 2e–16
Month February	0.4727498 (0.0339011)	< 2e–16	0.6179138 (0.0614459)	< 2e–16
Month January	0.5595071 (0.0360984)	< 2e–16	0.6190579 (0.0648127)	< 2e–16
Month July	0.2041098 (0.0314755)	8.89e–11	0.1460258 (0.0593064)	0.01381
Month June	0.2536605 (0.0316676)	1.15e–15	0.1937207 (0.0588766)	0.00100
Month March	0.5101808 (0.0326023)	< 2e–16	0.5079790 (0.0602246)	< 2e–16
Month May	0.2597362 (0.0312052)	< 2e–16	0.2216359 (0.0576237)	0.00012
Month November	0.7095393 (0.0297147)	< 2e–16	0.6977014 (0.0570473)	< 2e–16
Month October	0.3252624 (0.0310680)	< 2e–16	0.3767558 (0.0572062)	4.52e–11
Month September	0.4173954 (0.0312603)	< 2e–16	0.3839196 (0.0587822)	6.52e–11
April (Reference)				
Elevation > 2000 m	0.2372410 (0.0291927)	4.41e–16	0.1554760 (0.0537200)	0.00380
Elv1801-1900 m	0.3615582 (0.0236513)	< 2e–16	0.3888692 (0.0425264)	< 2e–16
Elv1901-2000 m	0.2054667 (0.0237197)	< 2e–16	0.1932937 (0.0422989)	4.88e–06
Elv < 1800 m (Reference)				
Rainfall	0.0031166 (0.0001301)	< 2e–16	0.0031249 (0.0002604)	< 2e–16
TMP AVG	– 0.1036946 (0.0050472)	< 2e–16	– 0.1262568 (0.0099988)	< 2e–16
TMPMIN	0.0075262 (0.0040111)	0.0606	0.0071389 (0.0074662)	0.33899
Sex male	0.0518893 (0.0118691)	1.23e–05	0.1047463 (0.0228256)	4.45e–06
Female (Reference)				
Age 5–14	– 0.7515411 (0.0145588)	< 2e–16	– 0.7747979 (0.0275689)	< 2e–16
Age < 5	– 0.7798807 (0.0147003)	< 2e–16	– 0.8021734 (0.0276449)	< 2e–16
Age 15 + (Reference)				
Theta (std. error)	–		0.46420	
AIC	89,437		67,948	
BIC	89607.08		69211.15	

Multiple Negative Binomial analysis indicated that season, rainfall, elevation, mean temperature, sex, and age category were significantly associated with malaria incidence. November was more likely [exp (0.6977)] to have higher malaria incidence as compared to April. The least malaria incidence was recorded in April. For a unit increment of rainfall in millimetres the odds of malaria incidence increase by exp (0.0031249) = 1.003 (adjusted for the other variables), which implies that for a unit increment of rainfall malaria incidence increases by 0.3%. Elevation 1801–1900 m equals exp (0.3888692) = 1.475 (adjusted for the other variables), which implies that malaria incidence on elevation 1801–1900 m was 1.475 times more likely observed than elevation > 2000 m. For a unit increment of mean temperature, malaria incidence decreases [exp (-0.1263) = 0.88] by 11.86%. Malaria incidence in males was 1.11 times more likely than in females. Malaria incidence in the age group of 5–14 years was [exp (-0.7748)] 0.4608 times less likely than the age group of 15 and above years.

It is often interesting to compare these models in a statistical sense using the Akaike information criterion (AIC) and Bayesian Information Criteria (BIC). In much literature reviewed that the models with the smallest AIC and BIC value is the better one and therefore, Negative Binomial regression model is responsible model to account for this data (Table 5).

## Discussion

Globally, the burden of malaria is declining due to the intensive and wide range implementation of preventive and control measures, and many countries are heading to achieve the elimination goal by 2030. However, several factors could challenge and reverse or delay the success of the elimination programme [5, 6]. In the current study, the effect of meteorological factors and altitude on malaria incidence was investigated. Accordingly, the finding revealed that rainfall, temperature, and elevation at the kebele/village levels were the major drivers of the disease.

In general, monthly rainfall and temperature at specific time lags had influenced the incidence of malaria. Rainfall had a positive correlation with malaria occurrences at lag 0 and lag 2 months. This indicated the increased risk of malaria transmission during the rainy time and when it gets lower at the end of the 2nd -month lag. Similar findings were also reported from different areas of the country and elsewhere in the world. A study in Kenya reported an increased incidence of malaria with 2-month lags of rainfall. A study in southwest Ethiopia reported that mean rainfall has a positive correlation with the malaria incidence at lag two to four months. Similarly, rainfall was a direct driver of malaria in Eritrea with various lag times (Lag 1, 2, 3, and 4) of the month. In addition, a strong correlation between rainfall and malaria transmission was reported from different geographical settings of Africa [19, 26, 27]. However, contrary, a negative association was reported from West Africa [16], whereas an insignificant association was reported from Ghana [28].

The lag effect of temperature was observed on the burden of malaria incidence in the current study. As a result, the minimum temperature showed a negative correlation with malaria incidence. A similar finding was reported from Ghana where minimum temperature lagged at three months was negatively correlated with malaria incidence. This could be due to the temperature that has a critical role in the regulation of growth, development, and survivor-ship of mosquito and malaria parasite, and also determines the period of the gonotrophic cycle. Hence, a minimum temperature could impede the development of mosquitoes’ larvae and pupae. As a result, a minimum temperature could delay the infectiousness of mosquitoes and results in low malaria transmission [28].

In this study, the maximum temperature was also negatively correlated with malaria incidence. A similar finding was also reported from southwest Ethiopia, where the maximum temperature at several lags, Lag 0 to 4 months was negatively correlated with the malaria incidence. A negative association of temperature and malaria incidence was also reported from Western Ethiopia [16, 19]. In contrast, others reported the positive correlation of temperature with malaria incidence. On the other hand, there was no association between monthly malaria incidence and mean maximum temperature in India. Besides, the influence of meteorological variables varies in different settings of the same country. For instance, rainfall, minimum, and maximum temperature showed a different correlation with malaria incidence in different regions of Ethiopia [29, 30].

These findings depicted the complex and multi-factorial epidemiology of malaria, which in turn requires a thorough investigation of different approaches. Also, the meteorological factor is not the only factor influencing malaria incidences. Other factors such as land-use, land cover, water management, populations at risk, demographic and socioeconomic status of a community, poor hygiene, population movement, and activities exposing to infection and utilization of existing interventions could highly determine the transmission pattern. Besides, several models have been used in different studies thus the findings require precautions during interpretations [8, 9, 28, 29, 31–33]. Therefore, further investigation of potential determinants of malaria transmission is highly recommended to device evidence-based intervention of malaria elimination.

Elevation has impacted the incidence of the disease. Thus, as altitude increased the burden of disease was decreased. As the altitude increase the temperature decrease, and vice versa that influences the incidence and transmission of infection [10–12]. In addition, the geographical shifts of the *Anopheles* mosquito due to climate change facilitate malaria transmission in previously non-malarious areas [13, 14], and the variation of the range of temperature influences the incidence and transmission of infection due to its direct effect on development and survivorship of vectors and malaria parasites [8]. Furthermore, altitude has an indirect effect on malaria transmission by determining temperature. As the altitude increase the temperature decrease, and vice versa that influences incidence and transmission [10–12].

The predictive tool was developed to forecast malaria incidence in the future, which is highly useful to track the pace of elimination for the reasonable allocation of a scarce resource. As a result, the ARIMA (2, 1, 2) model, the best fit model was used to predict the malaria incidence by 2030, the target year for malaria elimination. Accordingly, a point forecasts for the year 2030 shows that the number of malaria case will fluctuate around 88 per month, which is approximately constant throughout the year. This showed the probable reduction of malaria incidence and indicated whether the elimination goal would be achieved in a defined time or not.

Similarly, according to global modelling of malaria eradication trajectory for 2030 and 2050, the burden of malaria will increase in some parts of America and the Horn of Africa due to rising temperature and precipitation. In addition, as of the recent trajectory, expected global technical strategy milestones of malaria incidence set for 2020, 2025, and 2030 will not be achieved. This is because, given estimated 57 malaria incidence per 1000 population at risk in 2018, estimated malaria incidence in 2020, 2025, and 2030 would be 54, 48, and 42 instead of the required 34, 14, and 6 per 1000 population at risk, respectively, to meet the milestones of global technical strategy for malaria [4, 6].

Although the malaria incidence has been declined in the past decades the number of cases from 2015 to 2017 warranted the possible resurgence of the disease. Besides, 2% more malaria case was reported in 2016 compared to 2015, which raised questions whether global malaria elimination programme could be attained on time or not [4, 5]. Therefore, the prospect of malaria elimination requires more effort and attention than ever. Therefore, efficient and effective utilization of existing interventions, new alternative and complementary control intervention technologies/tools, and allocating/mobilizing adequate resources is highly required to achieve the elimination goal during the defined period.

In this study, other meteorological parameters like relative humidity, the effect of malaria interventions and entomology information were not included, which is the limitation of the study in general and a prediction model in particular.

## Conclusions

The findings of the present study showed a significant effect of rainfall and temperature on malaria incidence at several lags of the month. Rainfall was positively correlated with the malaria incidence while the temperature was negatively correlated. In addition, there was a variation of malaria distribution across different levels of geographical elevation. These were among the primary determinants of malaria incidence while other factors, such as malaria interventions and the abundance of malaria vectors could also influence the incidence of malaria. Therefore, a further study addressing such factors is required for further understanding and will be the next prior assignment in the study setting. Besides, equitable distribution and effective utilization of the existing malaria interventions are highly required to attain the elimination goal of the country.

## Data Availability

All data underlying the findings are available from corresponding authors on reasonable request.

## Abbreviations

WHO: World Health Organization
NMA: National Meteorological Agency
RDT: Rapid diagnostic test
ARIMA: Autoregressive Integrated Moving Average
GLM: General Linear Model
ACF: Auto-correlation function
PACF: Partial autocorrelation function
AIC: Akaike’s information Criteria
BIC: Bayesian information Criteria

## References

[1] Cibulskis RE, Alonso P, Aponte J, Aregawi M, Barrette A, Bergeron L (2016). Malaria: global progress 2000–2015 and future challenges. Infect Dis Poverty.

[2] WHO (2015). Global Technical Strategy for Malaria 2016–2030.

[3] WHO (2017). A framework for malaria elimination.

[4] Feachem RGA, Chen I, Akbari O, Bertozzi-Villa A, Bhatt S, Binka F (2019). Malaria eradication within a generation: ambitious, achievable, and necessary. Lancet.

[5] Dhiman S (2019). Are malaria elimination efforts on right track? An analysis of gains achieved and challenges ahead. Infect Dis Poverty.

[6] WHO (2019). World Malaria Report.

[7] Rossati A, Bargiacchi O, Kroumova V, Zaramella M, Caputo A, Garavelli PL (2016). Climate, environment and transmission of malaria. Infez Med.

[8] Fouque F, Reeder JC (2019). Impact of past and on-going changes on climate and weather on vector-borne diseases transmission: a look at the evidence. Infect Dis Poverty.

[9] Cella W, Baia-da-Silva DC, de Melo GC, Tadei WP, Sampaio VS, Pimenta P (2019). Do climate changes alter the distribution and transmission of malaria? Evidence assessment and recommendations for future studies. Rev Soc Bras Med Trop.

[10] Maxwell CA, Chambo W, Mwaimu M, Magogo F, Carneiro IA, Curtis CF (2003). Variation of malaria transmission and morbidity with altitude in Tanzania and with introduction of alphacypermethrin treated nets. Malar J.

[11] Dhiman RC, Yadav YK, Saraswat S (2013). SinghP. Altitude, temperature, and malaria vectors in Nainital and Udham Singh Nagar districts of Uttarakhand, India: An evidence-based study. J Vector Borne Dis.

[12] Guerra CA, Snow RW, Hay SI (2006). Defining the global spatial limits of malaria transmission in 2005. Adv Parasitol.

[13] Dhimal M, Ahrens B, Kuch U (2014). Altitudinal shift of malaria vectors and malaria elimination in Nepal. Malar J.

[14] Afrane YA, Githeko AK, Yan G, Casalegno S (2011). Malaria transmission in the African highlands in a changing climate situation: perspective from Kenyan highlands. Global warming impacts: Case studies on the economy, human health, and on urban and natural environments.

[15] Pawar A, Kumar S (2017). An analytical review on inter-relationships between climate change and malaria transmission. WNOFNS.

[16] Arab A, Jackson MC, Kongoli C (2014). Modelling the effects of weather and climate on malaria distributions in West Africa. Malar J.

[17] Loha E, Lindtjørn B (2010). Model variations in predicting incidence of *Plasmodium falciparum* malaria using 1998–2007 morbidity and meteorological data from south Ethiopia. Malar J.

[18] Kassa AW, Beyene BB (2014). Climate variability and malaria transmission – Fogera district, Ethiopia, 2003–2011. Sci J Public Health.

[19] Sena L, Deressa W, Ali A (2015). Correlation of climate variability and malaria: a retrospective comparative study, Southwest Ethiopia. Ethiop J Health Sci.

[20] Ministry of Health (2014). An epidemiological profile of malaria in Ethiopia.

[21] Southern Nations Nationalities and Peoples’. Regional State Finance and Economic Development Bureau. Annual Performance Report 2018. Hawassa, Ethiopia.

[22] Sidama Zone Agriculture Department. Annual Report. Hawassa, Ethiopia; 2018.

[23] Sidama Zone Health Department. Annual Report. Hawassa, Ethiopia; 2018.

[24] National Meteorology Agency. Monthly Records of Meteorology Data from 2010 to 2017. Addis Ababa, Ethiopia; 2020.

[25] Ministry of Health. National malaria guidelines fourth edition. Addis Ababa; 2018.

[26] Kipruto EK, Ochieng AO, Anyona DN, Mbalanya M, Mutua EN, Onguru D (2017). Effect of climatic variability on malaria trends in Baringo County, Kenya. Malar J.

[27] Kifle MM, Teklemariam TT, Teweldeberhan AM, Tesfamariam EH, Andegiorgish AK, Kidane EA (2019). Malaria risk stratification and modeling the effect of rainfall on malaria incidence in Eritrea. J Environ Public Health.

[28] Darkoh EL, Larbi JA, Lawer EA (2017). A weather-based prediction model of malaria prevalence in Amenfi West District. Ghana. Malar Res Treat..

[29] Kumar V, Mangal A, Panesar S, Yadav G, Talwar R, Raut D (2014). Forecasting malaria cases using climatic factors in Delhi, India: a time series analysis. Malar Res Treat.

[30] Taye G, Kaba M, Woyessa A, DeressaW, Simane B, Kumie A (2015). Modeling effect of climate variability on malaria in Ethiopia. Ethiop J Health Dev.

[31] Ferrão JL, Mendes JM, Painho M (2017). Modelling the influence of climate on malaria occurrence in Chimoio Municipality, Mozambique. Parasit Vectors.

[32] Mabaso MLH, Ndlovu NC (2012). Critical review of research literature on climate-driven malaria epidemics in sub-Saharan Africa. Public Health.

[33] Lindtjørn B, Loha E, Deressa W, Balkew M, Gebremichael T, Sorteberg A (2014). Strengthening malaria and climate research in Ethiopia. Malar J.

